# Evaluating the Role of Antibiotics in Patients Admitted to Hospital With Decompensated Cirrhosis: Lessons From the ATTIRE Trial

**DOI:** 10.14309/ajg.0000000000001937

**Published:** 2022-08-12

**Authors:** Rishen Kutmutia, Thais Tittanegro, Louise China, Ewan Forrest, Yiannis Kallis, Stephen D. Ryder, Gavin Wright, Nick Freemantle, Alastair O'Brien

**Affiliations:** 1 Institute of Liver and Digestive Health, University College London, London, UK;; 2 Glasgow Royal Infirmary, Glasgow, UK;; 3 Barts and the London School of Medicine and Dentistry Queen Mary University of London, London, UK;; 4 National Institute for Health Research Nottingham Biomedical Research Centre at Nottingham University Hospitals NHS Trust and the University of Nottingham, Queens Medical Centre, Nottingham, UK;; 5 Mid and South Essex NHS Foundation Trust, Basildon & Thurrock University Hospitals NHS Foundation Trust, Honorary Consultant in Gastroenterology, Hepatology and Hepatobiliary Medicine, The Royal Free Hospital, Honorary Senior Lecturer, University College London, Honorary Senior Clinical Lecturer, Kings College London, London, UK;; 6 Comprehensive Clinical Trials Unit, University College London, London, UK.

## Abstract

Hospital-acquired infections (HAI) are common in cirrhosis with antibiotics frequently used to prevent infections, but their efficacy for this role is unknown. To investigate this, we used Albumin to Prevent Infection in Chronic Liver Failure (ATTIRE) data to evaluate whether antibiotic use in patients without infection prevented HAI.

**METHODS:**

In ATTIRE patients without infection at baseline grouped by antibiotic prescription or not, we studied HAI during trial treatment period and mortality, with propensity score matching to account for differences in disease severity.

**RESULTS:**

Two hundred three of 408 patients prescribed antibiotics at enrollment did not have infection and they were more unwell than noninfected patients not given antibiotics. There were no differences in subsequent HAI comparing antibiotic treated (39/203, 19.2%) to nonantibiotic treated (73/360, 20.3%; *P* = 0.83). Twenty-eight-day mortality was higher in antibiotic-treated patients (*P* = 0.004) likely reflecting increased disease severity. Matching groups using propensity scoring revealed no differences in HAI or mortality. In noninfected patients at enrollment treated with/without rifaximin, there were no differences in HAI (*P* = 0.16) or mortality, confirmed with propensity matching. Patients given long-term antibiotic prophylaxis at discharge had no differences in 6-month mortality compared with nonantibiotic patients, although antibiotic-treated patients had more infections at trial entry, with numbers too small for matching.

**DISCUSSION:**

Half of antibiotics at study entry were given to patients without an infection diagnosis which did not reduce the overall risk of HAI or improve mortality. This supports prompt de-escalation or discontinuation of antibiotics guided by culture sensitivities at 24–48 hours after commencement if no infection and the patient is improving.

## INTRODUCTION

Infections in patients hospitalized with decompensated cirrhosis are common with a high mortality (1,2). Therefore, guidelines support early antibiotic prescription in suspected infection as mortality risk increases in (noncirrhotic) septic patients after delay in appropriate antibiotic therapy (3,4). Given this increased risk of infections, clinicians also prescribe broad-spectrum antibiotics to patients hospitalized with cirrhosis without infection to prevent hospital-acquired infections (HAI) and subsequent sepsis, as we observed in the Albumin to Prevent Infection in Chronic Liver Failure (ATTIRE) trial with 50% receiving antibiotics at trial entry, although only 27% were diagnosed with infection (5).

Other approaches to prevent infection may potentially include use of rifaximin in outpatients, a nonabsorbable broad-spectrum antibiotic licensed for hepatic encephalopathy (HE) (6–8), because this may prevent spontaneous bacterial peritonitis (SBP) (9). However, not all studies have shown benefit (10–12). Long-term antibiotics are also used in outpatients with ascites to prevent infection after an episode of SBP (13–15), supported by a 72% reduction in 3-month mortality (16), although a Cochrane systematic review concluded there was very low-certainty supportive evidence (17).

However, indiscriminate antibiotic use drives antimicrobial resistance (AMR) (18), and quinolone prophylaxis has been associated with increased multidrug resistance and Gram-positive organisms cultured in SBP (19–21). Patients hospitalized with cirrhosis have a high incidence of AMR with 34% of cultures growing multidrug-resistant organisms in a global study which caused higher mortality than non-AMR infections (22). Therefore, prescribing antibiotics in the absence of infection, which may actually be harmful in the longer-term and judicious use of antibiotics, is a global priority (18). More evidence is needed to aid decision-making on the use of antibiotics to prevent infection in decompensated cirrhosis, in particular in hospitalized patients (23,24). We therefore estimated the risk of HAI and mortality in patients without active infection at hospitalization who were treated with antibiotics or rifaximin compared with those who were not treated, using data collected from our completed ATTIRE trial (5).

## METHODS

### ATTIRE trial

ATTIRE was a neutral trial of targeted albumin infusions versus standard care involving 777 patients hospitalized with decompensated cirrhosis from 35 hospitals across England, Wales, and Scotland (2016–2019).

### Primary analysis for the current study

We compared incidence of HAI on days 3–15 between patients prescribed antibiotics (except rifaximin) at baseline (trial entry) and not, who were categorized as *not* having a clinical diagnosis of infection at baseline.

#### Data collection.

Baseline assessment included clinician (attending team) diagnosis of infection and antibiotic prescription. Data were collected daily until discharge, death, medically fit for discharge, or day 15. Incidence of any infection from days 3–15 was part of ATTIRE'S primary composite end point, which we categorized as HAI as onset >48 hours after hospitalization.

#### Statistical analysis.

Three of 777 participants had missing antibiotic status and were excluded. Antibiotic treatment was extracted from the concomitant medication case report forms (CRF). Data included name, dose, and start/stop date for all medications during the trial from drug charts and were inputted into the ATTIRE database at the UCL Comprehensive Clinical Trials Unit.

Infection diagnosis in decompensated cirrhosis can be difficult, with a high incidence of negative microbial cultures (25). ATTIRE defined infection according to attending clinician's diagnosis, and sites were then asked to complete infection CRFs with supporting clinical, biochemical, microbiological, and radiological data. These were blindly scrutinized by a panel of 3 physicians to categorize information provided as making infection diagnosis likely or unlikely. Physicians were asked to follow their normal clinical practice for diagnosis and management of infection. For this substudy, we used these data to investigate any systematic differences in HAI diagnoses being considered unlikely according to baseline antibiotics treatment or not.

#### Secondary outcomes.

Secondary outcomes were mortality to 6 months from trial entry and renal dysfunction during the trial treatment period, defined as a serum creatinine increase ≥50% from randomization, or patient initiated on renal replacement, or rise in creatinine ≥26.5 μmol/L within 48 hours. We reported these from day 3 onward as infection.

#### Propensity scoring.

Propensity scoring was used to account for baseline differences in disease severity, when numbers were adequate to provide a robust basis for estimation, defined prospectively as ≥50. We calculated a propensity score for each subject, using the fitted value on the logit scale from a logistic regression model which included baseline variceal bleed, new-onset/worsening ascites, HE, gender, age, Model for End-Stage Liver Disease (MELD) score, serum albumin, creatinine, white cell count (WCC), C-reactive protein (CRP), and randomized group. Explanatory variables in the baseline model were modified until an adequate match was achieved between cases and controls assessed by relevant standardized mean differences. Cases and controls were matched on propensity scores using a nearest neighbor matching procedure without replacement, with a caliper width of 0.01 on the logit scale. The matched data set was locked before proceeding to preplanned outcome analyses (26,27).

#### Subgroup analyses.

##### Excluding patients with acute variceal hemorrhage.

As prophylactic antibiotics have shown benefit and are widely used in these patients (28).

##### Patients with increased systemic inflammation at hospitalization but no infection.

Patients hospitalized with cirrhosis with elevated systemic inflammation, without infection, have an increased risk of HAI and mortality (29,30) and so we examined whether prophylactic antibiotics reduced HAI in patients with elevated WCC and no infection. As a normal range for WCC is 4–11 × 10^9^/L (31), we used WCC >10.99 to create a binary column indicating 1 when WCC ≥11.

### Other analyses

#### Use of rifaximin in patients without infection at baseline.

We examined whether patients without baseline infection and taking rifaximin before hospitalization had reduced HAI, less renal dysfunction, and improved survival compared with those not. To distinguish between patients taking rifaximin before admission and those commenced during hospitalization, we only considered entries with a start date before or at randomization.

For those taking rifaximin at discharge, we included patients with prescription start but no end date to exclude prescriptions stopped during hospitalization and examined 6-month mortality.

#### Patients taking long-term antibiotic prophylaxis before hospitalization and at discharge.

We examined infection at baseline, HAI, and 6-month mortality in patients identified as taking long-term antibiotic prophylaxis before hospitalization. To distinguish prophylaxis from treatment, we took advantage of dosing for long-term prophylaxis being oral and lower than treatment and searched for oral ciprofloxacin 250 mg once or twice a day (od/bd) or 500 mg once a day, oral co-trimoxazole 960 mg once a day, oral norfloxacin 500 mg od (none), and augmentin 375/625 mg od (none). We only considered entries with a start date before or at randomization to select those taking prophylaxis before hospitalization. Patients taking antibiotics intravenously, of higher doses, with concomitant antibiotics (e.g., ciprofloxacin and metronidazole), or >2 days after enrollment were considered treatment, not prophylaxis. We identified 11 patients taking both rifaximin and antibiotic prophylaxis before hospitalization, which is too small for additional analyses.

For prophylaxis at discharge, we included patients with prescription start but no end date to exclude prescriptions stopped during hospitalization. We also identified patients commenced on new prophylaxis after at least 5 days of intravenous antibiotics (for treatment) before prophylaxis commended (and no end date for prophylaxis prescription). We examined 6-month mortality.

A statistical analysis plan, before analyses, was approved by all authors. All authors vouch for completeness and accuracy of data. Data were not adjusted for multiple comparisons. Microsoft Excel was used for extraction of data from ATTIRE databases and producing tables.

IBM SPSS version 27 was used for bivariate tests of statistical significance (*t* tests for continuous variables and χ^2^ tests for categorical variables). Other analyses were performed using SAS software, version 9.4 (SAS Institute, Cary, NC).

## RESULTS

### Incidence of HAI in patients prescribed antibiotics or not in the absence of a diagnosis of infection at ATTIRE trial entry

We identified 563 patients without infection at trial entry with 203 of 563 (36%) commenced on antibiotics (see Supplementary Figure 1, Supplementary Digital Content 1, http://links.lww.com/AJG/C616). Patients without a diagnosis of infection accounted for 49.8% of all those started on antibiotics at baseline (203/408). These 203 noninfection patients were given 242 separate prescriptions, administered in total for 1,042 days. The most common were piperacillin–tazobactam (81, 33%), co-amoxiclav (61, 25%), and ciprofloxacin (23, 9.5%) (see Supplementary Table 1, Supplementary Digital Content 1, http://links.lww.com/AJG/C616).

Patients prescribed antibiotics without infection had similar age, gender, presence of ascites, and serum albumin to those not, but had significantly increased variceal bleeds, HE, creatinine, WCC, and CRP (Table 1a). HAI during the trial were similar in those prescribed antibiotics at baseline, 39 of 203 (19.2%), compared with not, 73 of 360 (20.3%) (*P* = 0.83). Twenty-eight-day mortality was significantly higher in antibiotic-treated patients (*P* = 0.004), but not 3- or 6-month mortality. Renal dysfunction during the trial treatment period was similar between groups.

**Table 1.**
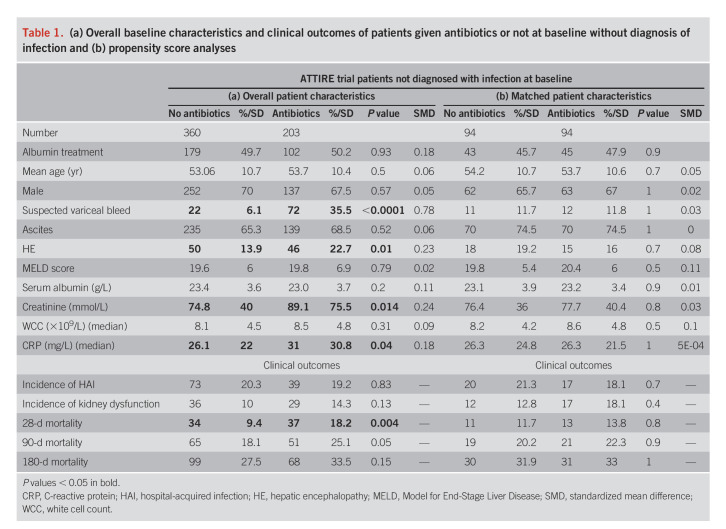
(a) Overall baseline characteristics and clinical outcomes of patients given antibiotics or not at baseline without diagnosis of infection and (b) propensity score analyses

#### Propensity score–matched analysis.

Patients prescribed antibiotics in the absence of infection were more unwell than those not, and we undertook propensity score matching to account for this. Propensity scores were calculated for patients not diagnosed with infection at trial entry. One hundred eighty-eight were matched (94 given antibiotics matched individually with 94 not, Table 1b). This was successful with no significant differences between groups for baseline characteristics and low standardized mean differences between matched cohorts. Analyses showed no differences in HAI with 17 of 94 (18.1%) infections in the antibiotic group and 20 of 94 (21.3%) in nonantibiotic (*P* = 0.71). There were no differences in mortality nor renal dysfunction during the trial between groups.

Collectively, these data show no overall reduction in HAI in patients prescribed antibiotics at baseline in the absence of a clinical diagnosis of infection. As antibiotics are routinely prescribed for acute variceal bleeding irrespective of infection diagnosis, we next excluded these patients from our analyses.

#### Excluding patients with acute variceal hemorrhage at baseline.

We excluded 115 patients with variceal hemorrhage and 11 with lack of data (unknown antibiotics [3] or variceal hemorrhage [8]). One hundred twenty-nine antibiotic-treated patients were compared with 334 nonantibiotic (Table 2). Groups had similar ages, gender, MELD score, and serum albumin. Again, those prescribed antibiotics had higher prevalence of ascites and HE and higher creatinine, WCC, and CRP. Both had similar incidence of HAI, 28 of 129 (21.7%) in antibiotic-treated and 66 of 334 (19.8%) in nonantibiotic (*P* = 0.64). As expected in more severely unwell patients, mortality was significantly higher in antibiotic-treated patients at 28 and 90 days (*P* = 0.005 and 0.042, respectively) and there was a nonsignificant increase in renal dysfunction in antibiotic-treated patients.

**Table 2.**
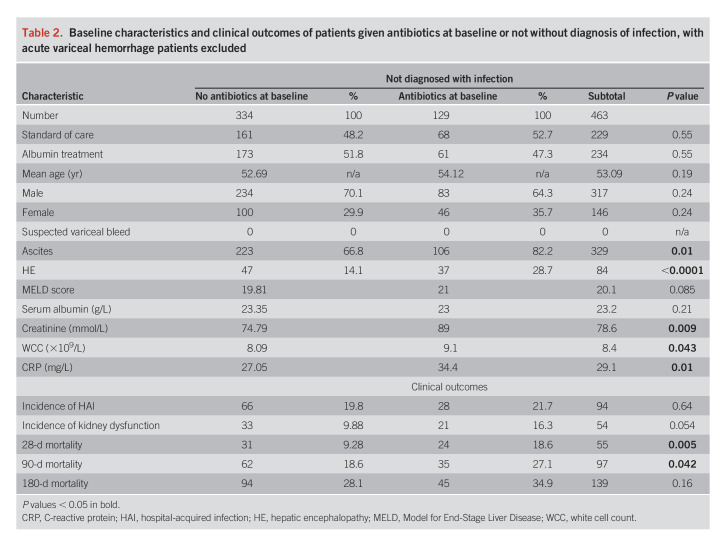
Baseline characteristics and clinical outcomes of patients given antibiotics at baseline or not without diagnosis of infection, with acute variceal hemorrhage patients excluded

#### Descriptive analysis of HAI.

There were no differences between groups in the independent panel's assessment of infection diagnosis as likely or unlikely according to CRF data, with 8 of 39 (21.6%) of HAI diagnoses in the baseline antibiotic group considered unlikely and 11 of 73 (15.1%) in the nonantibiotic group (*P* = 0.46).

The median day of overall HAI diagnosis was 6 with the most common infections such as respiratory (39%), SBP (10%), urinary tract infection (8%), and skin/soft tissue (8%). Of all 129 recorded CRF, 39 (30%) were microbial culture positive, with only 5 (10%) positive respiratory infections. The most common organisms were staphylococcal, enterococci, and *Escherichia coli* (7 each) with 4 *coliforms*. AMR was reported in 2 patients (1 *coliform* and 1 *Enterococcus faecium*), with 31 susceptible organisms and no data in 4 (see Supplementary Table 2, Supplementary Digital Content 1, http://links.lww.com/AJG/C616).

#### Patients with serum WCC ≥11 × 10^9^/L at baseline but no infection.

We identified 125 patients fulfilling criteria with 50 prescribed antibiotics. Once more, those prescribed antibiotics were more unwell with elevated creatinine, higher MELD, and more variceal bleeds. There were no significant differences in HAI, 10 of 50 (20%) infections in the antibiotic group and 19 of 75 (25%) in the nonantibiotic group (*P* = 0.55). There were no significant differences in mortality nor renal dysfunction during the trial between groups (Table 3a).

**Table 3.**
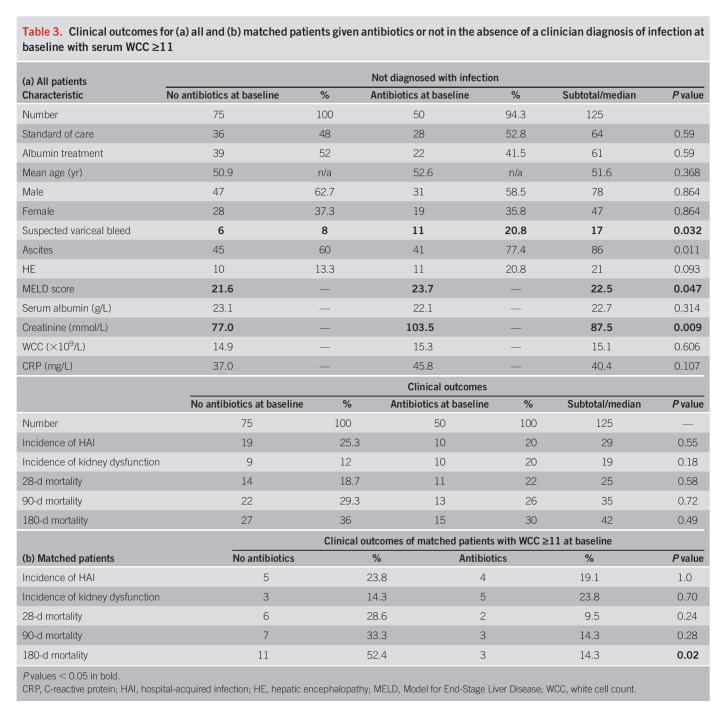
Clinical outcomes for (a) all and (b) matched patients given antibiotics or not in the absence of a clinician diagnosis of infection at baseline with serum WCC ≥11

Forty-two subjects with WCC ≥11 × 10^9^ from original matching were identified (21 cases and controls). There were no differences between HAI, 4 of 21 (19.1%) in antibiotic treated and 5 of 21 (23.8%) in nonantibiotic (*P* = 1), nor renal dysfunction. The difference in mortality between antibiotic and nonantibiotic groups increased over time, until statistically significant at 180 days (*P* = 0.02) (Table 3b).

### Incidence of HAI in patients prescribed rifaximin or not without infection at ATTIRE trial entry

We identified 67 noninfected patients taking rifaximin at baseline and 497 not taking rifaximin. HE was 3 times higher in patients taking rifaximin (Table 4a). There were no significant differences in HAI, 10 of 67 (14.9%) in the rifaximin group and 102 of 497 (20.5%) in the nonrifaximin group (*P* = 0.16). There were no significant differences in 6-month mortality, nor renal dysfunction during the trial. Thirty-three pairs were propensity matched, with no differences in HAI (4/33 in rifaximin and 8/33 in nonrifaximin, *P* = 0.34), 6-month mortality, nor renal dysfunction (Table 4b).

**Table 4.**
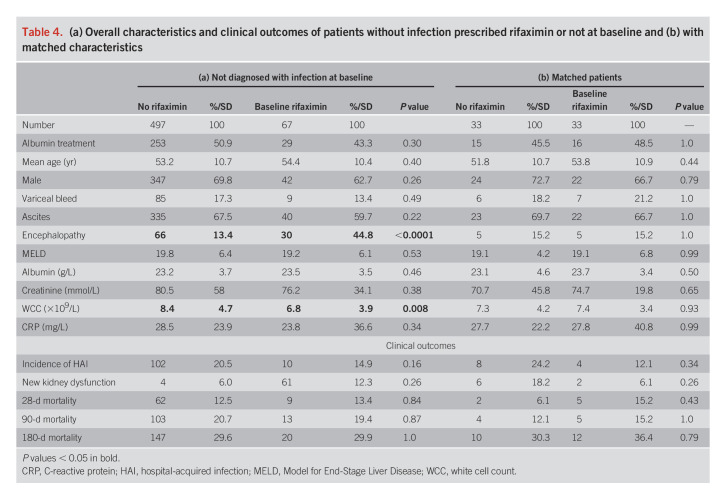
(a) Overall characteristics and clinical outcomes of patients without infection prescribed rifaximin or not at baseline and (b) with matched characteristics

We identified 79 patients at discharge taking rifaximin without a baseline infection diagnosis (Table 5). When compared with 442 alive at discharge with no baseline infection not taking rifaximin, we found substantial differences in baseline HE, creatinine, and CRP, but 6-month mortality was similar. Propensity matching in 35 pairs showed no significant differences in time-to-event for mortality (hazard ratio 1.31; 95% confidence interval 0.49–3.51; *P* = 0.595) (Table 5b and Supplementary Figure 2, Supplementary Digital Content 1, http://links.lww.com/AJG/C616).

**Table 5.**
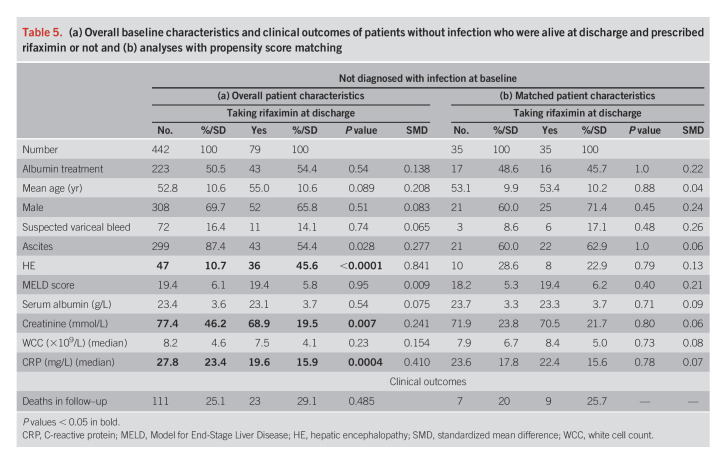
(a) Overall baseline characteristics and clinical outcomes of patients without infection who were alive at discharge and prescribed rifaximin or not and (b) analyses with propensity score matching

### Incidence of infection and mortality in ATTIRE trial patients taking long-term antibiotic prophylaxis or not before hospitalization and at discharge

Thirty-one patients were taking ciprofloxacin (20) or co-trimoxazole (11) prophylaxis at baseline. There were no significant differences in baseline infection rates between those taking prophylaxis, 5 of 30 (16.7%), or not, 206 of 747 (27.6%) (*P* = 0.16), nor differences in HAI or 6-month mortality (see Supplementary Table 3, Supplementary Digital Content 1, http://links.lww.com/AJG/C616).

Sixty-three were taking prophylaxis at discharge with 35 commenced on new prophylaxis (23 ciprofloxacin and 12 co-trimoxazole). Patients given long-term antibiotic prophylaxis at discharge had significantly increased infections at trial entry compared with nonantibiotic patients, but there were no significant differences in 6-month mortality between groups (*P* = 0.44 and *P* = 0.21, respectively, see Supplementary Tables 4 and 5, Supplementary Digital Content 1, http://links.lww.com/AJG/C616). Numbers were too small for matching.

## DISCUSSION

Half of all antibiotics prescribed to patients hospitalized with decompensated cirrhosis at ATTIRE trial entry were given to patients who were not diagnosed with infection and this accounted for greater than 1,000 prescription days for 203 patients. These patients were more unwell than those without infection who were not given antibiotics and we assumed prescription was aimed at reducing incidence of HAI in those considered at high risk. Yet overall, this approach did not reduce the risk of HAI during hospitalization nor improve mortality, with similar results seen when accounting for differences in disease severity between groups using propensity scores. This pattern was maintained when patients with acute variceal hemorrhage were excluded from analyses. The infection CRF revealed no bias in HAI reporting accuracy between antibiotic and nonantibiotic groups. There may be a mortality benefit for antibiotic use in patients without a diagnosis of infection but elevated WCC, although incidence of HAI was no different to those not given antibiotics and numbers were small. Use of rifaximin in patients without infection at baseline did not reduce HAI nor improve 6-month mortality, supported by propensity matching.

The rate of positive microbial culture was low at 30%, perhaps related to the most common infections being respiratory and the well-known difficulties collecting and obtaining pathogenic organisms from sputum (32). The very low AMR rate was encouraging but differs from the latest UK Health Security Agency data showing that 1 of 5 bloodstream infections overall were antibiotic-resistant (33) and data from patients with cirrhosis in other countries (22). Microbial culture data collection was not mandated, as opposed to clinician diagnosis of infection which formed part of the primary end point, and so data are likely an underestimate. Nevertheless, debate over the true current prevalence of AMR in cirrhosis within the UK does not lessen the importance of preserving antibiotics for future use.

Early aggressive empirical antibiotic treatment is widely advocated to combat the risk of infection in cirrhosis (34), and it is likely that the main motivating factor underlying the large numbers treated with antibiotics, despite no infection, was to prevent infection and sepsis in patients considered at high risk. Yet, antibiotic overuse can cause harm, as a major driver of AMR (18). When compared with other chronic diseases, patients with cirrhosis have increased hospitalizations, longer stays, more invasive procedures, and readmissions, all increasing AMR risk (35). This heightens the need to reduce unnecessary antibiotic prescriptions. Antibiotics reduce infections and mortality in acute variceal hemorrhage (28) and reduce infections, but not mortality, in severe alcoholic hepatitis (36) and acute-on-chronic-liver failure (37). However, in the baseline ATTIRE cohort, 14.8% had variceal hemorrhage, 10.6% creatinine ≥1.5 mg/dL, and only 2.3% were admitted to intensive care, and evidently, antibiotics were frequently used to prevent infections outside of existing evidence.

We found no evidence to support extending rifaximin's indication to prevention of infection in decompensated cirrhosis. We also observed no significant improvement in mortality in patients given long-term antibiotic prophylaxis at discharge, but these patients had more frequent incidence of infection at hospitalization and might therefore have been expected to have increased mortality. Consequently, prophylaxis may be beneficial, but numbers were too small to investigate with propensity score matching. Our real-world data demonstrate the continued uncertainty around management of infection in decompensated cirrhosis emphasizing the need for clinicians to search diligently for evidence of this and for further clinical research to identify who might benefit from empirical/prophylactic antibiotic use and for improved molecular diagnostic approaches to infection diagnosis.

ATTIRE was a large national trial and likely to represent national practice. HAI diagnosis was completed to a high standard and the neutral outcome for albumin enabled pooling of all data. We performed propensity score matching to account for baseline differences, although this might not capture clinical intuition which can lead to confounding by indication. Limitations were that this was not a randomized comparison of antibiotics to nonuse and we did not comprehensively capture microbiology data; however, we detected no bias in infection reporting between groups. Patients anticipated to have a short hospital stay were not included and the numbers of patients admitted to intensive care unit were low and therefore our results cannot necessarily be extrapolated to these cohorts. We performed multiple tests and for some there were low numbers. There was a little difficulty achieving a match for rifaximin with some standardized mean differences >0.2. Only 4 patients were transplanted during 6-month follow-up, and we did not collect data on transplant listing, and it is possible that findings may have differed slightly if more of these patients had been included. We did not collect data on subsequent infections, ongoing alcohol consumption, or hospital readmissions after hospital discharge. Finally, there may be other high-risk groups that may benefit from antibiotics to prevent infection that we did not study.

To conclude, UK physicians commonly prescribe antibiotics to patients hospitalized with decompensated cirrhosis without an infection. These patients were more unwell than those not prescribed antibiotics, and this was likely to have been to prevent infections in patients considered at high risk of sepsis. However, our comprehensive and strikingly consistent analyses showed no overall beneficial impact on preventing HAI, nor renal dysfunction and no impact on survival. These data support a policy of prompt de-escalation or discontinuation of empirical antibiotics guided by culture sensitivities at 24–48 hours after commencement if no infection and an improving patient.

## CONFLICTS OF INTEREST

**Guarantor of the article:** Alastair O'Brien, MD, PhD.

**Specific author contributions:** Databases were created by L.C., R.K., and T.T. and verified by A.O.'B. R.K., T.T., and N.F. performed statistical analyses. A.O.'B. wrote manuscript first draft, with contributions from all authors.

**Financial support:** Funded by the Health Innovation Challenge fund awarded to A.O.'B. (Wellcome Trust and Department of Health and Social Care) HICF reference HICF-R8-439, WT grant number WT102568.

**Potential competing interests:** None to report.

**Trial registration numbers:** ATTIRE trial EudraCT number: 2014-002300-24 and International Standard RCT Number: 14174793. Research Ethics Committee Number: 15/LO/0104.

**Study Highlights**

**WHAT IS KNOWN**

✓ Patients with decompensated cirrhosis are at high risk of hospital-acquired infection (HAI) and in certain circumstances, such as acute variceal hemorrhage, antibiotic prescribing in the absence of an active infection diagnosis is beneficial.
✓ However, we face a global crisis of antimicrobial resistance, driven in part by antibiotic overprescribing and reducing unnecessary use is a global priority.

**WHAT IS NEW HERE**

✓ Nearly half of the antibiotics prescribed at study entry to the large-scale ATTIRE trial were given to hospitalized patients without a clinical diagnosis of infection.
✓ We found no evidence that this approach prevented development of HAI, nor mortality overall, although there may be a benefit in those with a serum white cell count >11.
✓ Other analyses found no evidence to support the use of rifaximin to prevent HAI.
✓ These data demonstrate an overall lack of efficacy for antibiotic prescribing in the absence of infection to prevent HAI in decompensated cirrhosis, despite this being common practice in the United Kingdom.
✓ Considering these data, we suggest that infection guidelines and care bundles should include: (i) Prompt de-escalation or discontinuation of empirically prescribed antibiotics guided by culture sensitivities at 24–48 hours after commencement if no infection and an improving patient. (ii) Restricting prophylaxis use to evidence-based indications. (iii) Greater evidence is needed to recommend rifaximin use to prevent HAI.
✓ Finally, our real-world data demonstrate the continued uncertainty around management of infection in decompensated cirrhosis emphasizing the need for further clinical research to identify who might benefit from empirical/prophylactic antibiotic use and improved molecular diagnostic approaches to infection diagnosis.

## Supplementary Material
