# Supplementary material for: Evaluating the Role of Antibiotics in Patients Admitted to Hospital With Decompensated Cirrhosis: Lessons From the ATTIRE Trial
Source: Am J Gastroenterol. 2022 Aug 12;118(1):105–13. doi: 10.14309/ajg.0000000000001937 (PMC9810015; doi:10.14309/ajg.0000000000001937)

**Supplementary Material**

Supplementary Material…………………………………………………………………………………….. ….1

†ATTIRE Trial Investigators: Group Authorship

Trial Steering Committee……………………………………………………………………………...2

Independent Data Monitoring Committee...………………………………………………………....2

Data centre at University College Comprehensive Clinical Trials Unit (UCL CCTU)………..….2

Trial Management Group……………………………………………………………………………………..…3

University College London Comprehensive Clinical Trials Unit...…………………………………….........3

Research Steering Committee…...…………………………………………………………………………….3

Microbiology and Adverse Event Review Panel……………………………………………………………...3

ATTIRE Site Investigators………………………………………………………………………………………3

ATTIRE Clinical Trial Sites………………………..…………………………………………………………….3

Trial Funding and Conduct………………………………………………………………………………….…..4

Acknowledgements.................................................................................................................................4

ATTIRE Study Protocol…………………..………………………………………………………………..........5

Supplementary Tables 1……...…………………………………………………………………………………6

Supplementary Table 2………………………………………………………………………………………….6

Supplementary Table 3………………………………………………………………………………………….7

Supplementary Table 4………………………………………………………………………………………….8

Supplementary Table 5……………………………………………………………………..…………………..9

Supplementary Figure 1……………………………………………………………………………………….10

Supplementary Figure 2……………………………………………………………………………………….11

**ATTIRE Trial Investigators: Group Authorship**

**Independent Data Monitoring Group**: Professor Dominique-Charles Valla* (CHAIR), Tim Clayton^k^ and Dr Vipul Jaraith^#^.

**Data centre at University College Comprehensive Clinical Trials Unit (UCL CCTU):** Kate Bennett^, Scott Bevan^ǁ^, James Blackstone**^‡^**, Kashfia Chowdhury^‡^, Zainib Shabir**^‡^** and Simon Skene^.

**Trial Steering Committee:** Professor Stephen Brett (CHAIR)^#^, John Crookenden (Patient Representative), Professor Shahid Khan^#^, Brennan Kahan†, Professor Graeme Alexander^§^, Professor Humphrey Hodgson^§^ and Professor Mike Murphy^¥^.

**Affiliations (United Kingdom unless stated):** *Service d'Hépatologie, Hôpital Beaujon, 100 boulevard Général Leclerc, 92118, Clichy, France. dominique.valla@aphp.fr; ^k^The London School of Hygiene and Tropical Medicine, Tim.Clayton@Lshtm.ac.uk; ^#^Western University and London Health Sciences Centre, Canada, vjairath@uwo.ca.

^Surrey Clinical Trials Unit, University of Surrey, c.bennetteastley@surrey.ac.uk and s.skene@surrey.ac.uk; Bristol Medical School, Bristol, s.bevan@bristol.ac.uk; and ^‡^Comprehensive Clinical Trials Unit, University College London, j.blackstone@ucl.ac.uk.

^#^Imperial College London/Imperial College NHS Trust, United Kingdom stephen.brett@imperial.ac.uk and shahid.khan@imperial.ac.uk; †MRC Clinical Trials Unit at UCL, b.kahan@ucl.ac.uk; ^§^Institute of Liver and Digestive Health, University College London, gja1000@doctors.org.uk and h.hodgson@ucl.ac.uk; and ^¥^University of Oxford, mike.murphy@nhsbt.nhs.uk.

**Trial Management Group**

Dr Louise China, Professor Ewan H Forrest, Dr Yiannis Kallis, Dr Jim Portal, Professor Stephen Ryder and Dr Gavin Wright.

**UCL CCTU**

Dr Ana Arbeloa del Moral, James Blackstone, Kashfia Chowdhury, Dr Ana Carolina Estevao, Rosie Hamilton, Ms Khadra Mohamoud and Dr Nicola Muirhead.

**Research Steering Committee**

Professor Mauro Bernardi (CHAIR), Paula Milton (Department of Health and Social Care representative) and Nicola Shepherd (Wellcome Trust representative).

**Microbiology and Adverse Event Review Panel**

Dr Indran Balakrishnan, Dr Mark McPhail, Dr Brian Hogan and Dr Jane Abbott.

**ATTIRE Site Investigators**

Professor Aftab Ala, Dr Richard Aspinall, Dr Andrew Austin, Dr C Lye Ch'ng, Dr Jeremy Cobbold, Dr Lynsey Corless, Dr Alexandra Daley, Professor Matthew Cramp, Dr Ahmed Elsharkawy, Dr Alex Evans, Prof Graham Foster, Dr Shaun Greer, Dr Mathis Heydtmann, Dr Coral Hollywood, Dr Peter Isaacs, Professor Rajiv Jalan, Dr Richard Keld, Dr Andrew King, Dr Stuart McPherson, Dr Judith Morris, Professor Jane Metcalf, Dr Richard Parker, Dr Janisha Patel, Dr Francisco Porraz-Perez, Dr Praveen Rajasekhar, Dr John Ramage, Dr Paul Richardson, Dr Dariush Sadigh, Dr Deepak Suri, Dr Esther Unit, Professor Sumita Verma and Dr Earl Williams.

**ATTIRE Clinical Trial Sites**

Basildon, Basingstoke, Berkshire, Birmingham, Blackpool, Bournemouth, Bristol, Brighton, Coventry, Derby, Durham, Glasgow RI, Glasgow QE, Glasgow RA, Gloucestershire, Heartlands, Hull, Leeds, Liverpool, Manchester, Newcastle, North Tees, North Tyneside, Nottingham, Oxford, Plymouth, Portsmouth, Royal Free, Royal London, South Tyneside, Southampton, Surrey, Swansea, Whittington and Wigan.

**Trial funding**

The work was funded by the Health Innovation Challenge fund (Wellcome Trust and Department of Health and Social Care) HICF reference HICF-R8-439, WT grant number WT102568 awarded to Alastair O'Brien.

This publication presents independent research commissioned by the Health Innovation Challenge Fund, a parallel funding partnership between the Department of Health and Wellcome Trust. The views expressed in this publication are those of the author(s) and not necessarily those of the Department of Health and Social Care or Wellcome Trust.

The 20% Human Albumin Solution used was taken from routine hospital stocks throughout the UK and the study was funded wholly by the Wellcome Trust and Department of Health and Social Care grant to Professor O’Brien.

**Trial Conduct**

ATTIRE was conducted and reported according to the protocol, the Medicines for Human Use (Clinical Trials) Regulations 2004, (amended 2006), the European Union Clinical Trials Directive (2001/20/EC) guidelines, the principles of the International Conference on Harmonisation Good Clinical Practice under oversight of the University College London Comprehensive Clinical Trials Unit (UCL CCTU) and provisions of the Declaration of Helsinki.

**Acknowledgements**

The authors acknowledge funding from the Wellcome Trust and Department of Health and Social Care, the National Institute for Health Research Clinical Research Network for providing research nurse support, the University College London Comprehensive Clinical Trials Unit for trial management activities and University College London for sponsoring the trial.

**ATTIRE Study Protocol**

**
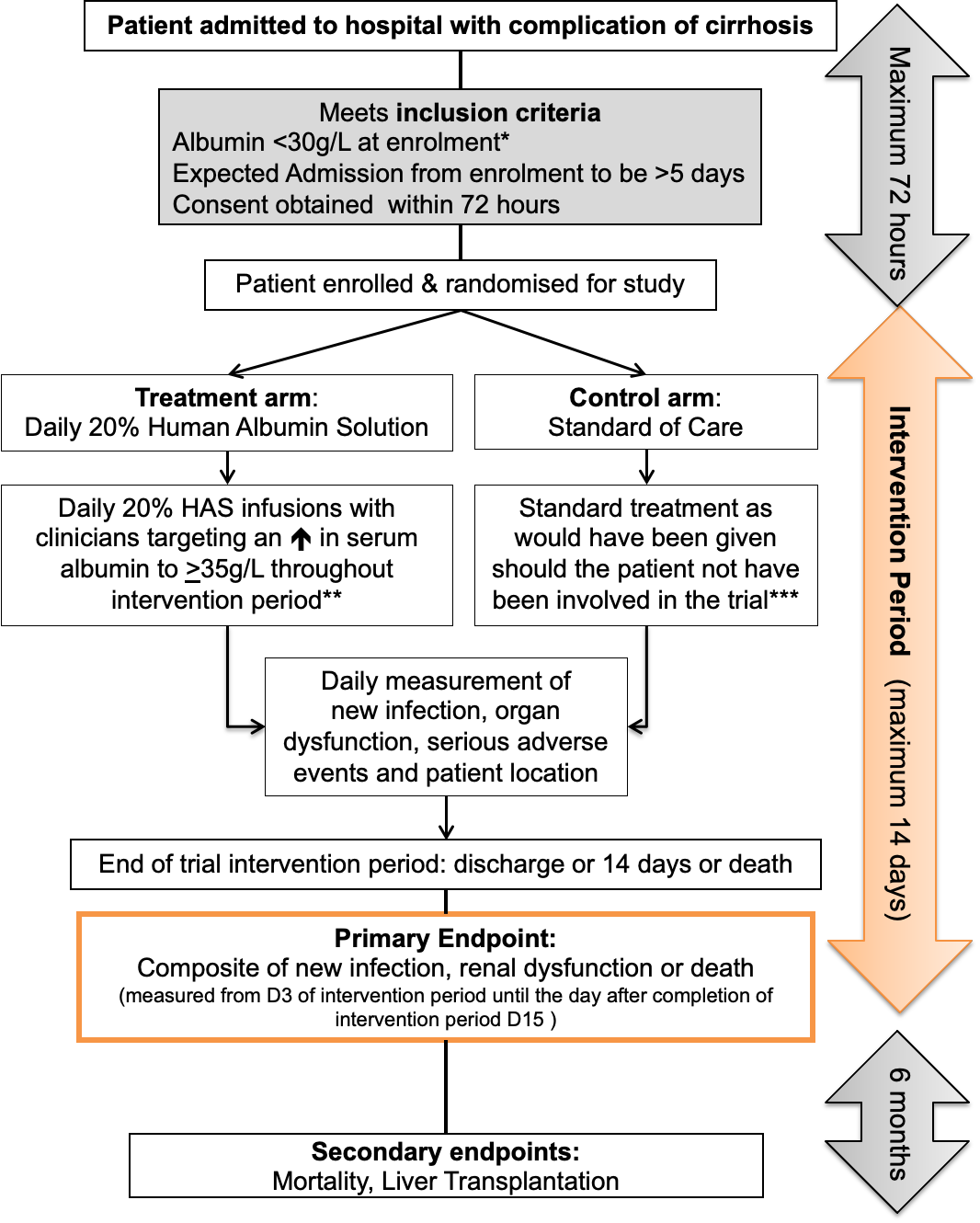
**

* Which could be any time point on days 1-3 (72 hours) of admission.

** See table S2 for infusion protocol. The study aim was for treatment arm patients to achieve and sustain a serum albumin >30g/L. This was achieved by asking site clinicians to target a serum albumin of >35g/L.

*** This can only include albumin as recommended in international evidence based guidance: LVP, SBP & HRS.

**Supplementary Table 1.** The antibiotics prescribed at ATTIRE trial baseline in patients without a clinical diagnosis of infection

| **Type of antibiotic prescribed** | **Number of prescriptions** |
| --- | --- |
| Piperacillin / Tazobactam | 81 |
| Co-amoxiclav | 61 |
| Ciprofloxacin | 23 |
| Ceftriaxone | 18 |
| Metronidazole | 16 |
| Amoxycillin | 11 |
| Cefotaxime | 9 |
| Co-Trimoxazole | 5 |
| Levofloxacin | 5 |

2 prescriptions each for Vancomycin, Clarithromycin, Cefuroxime, Teicoplanin and Trimethoprim.

1 prescription each for Flucloxacillin, Temocillin and Gentamicin.

**Supplementary Table 2.** Microbial organisms cultured for hospital acquired infections

| **Type of organism** | **Number of positive cultures** |
| --- | --- |
| E.coli | 7 |
| Enterococcus | 7 |
| Staphylococcus | 7 |
| Coliform | 4 |
| Gram positive cocci | 3 |
| Klebsiella | 2 |
| Streptococcus | 2 |

1 each for yeast, Clostridium difficile, Aspergillus, Gram negative bacillus, Acinetobacter, Enterobacter cloacae and Pseudomonas

**Supplementary Table 3.** Baseline characteristics and clinical outcomes of patients taking long term antibiotic prophylaxis or not prior to hospitalization (*P values* <0.05 in **bold)**.

|  | Prophylaxis at baseline | %/SD | Non-prophylaxis | %/SD | *P value* |
| --- | --- | --- | --- | --- | --- |
| Number | 30 | 100 | 747 | 100 | - |
| Mean age (yrs) | 56.5 | 10.5 | 53.7 | 10.6 | *.16* |
| Male | 24 | 80 | 525 | 70.3 | *.25* |
| Suspected Variceal Bleed | 6 | 20 | 109 | 14.6 | *.41* |
| Ascites | 24 | 80 | 493 | 66 | *.11* |
| Hepatic Encephalopathy | 8 | 26.7 | 141 | 18.9 | *.28* |
| Diagnosis of infection at randomization | 5 | 16.7 | 206 | 27.6 | *.19* |
| Use of antibiotics | 15 | 50 | 383 | 51.3 | *.89* |
| MELD Score | 17.9 | 5.2 | 19.6 | 6.3 | *.12* |
| Serum Albumin (g/L) | 24.5 | 3.3 | 24 | 3.7 | *.08* |
| Creatinine (mmol/L) | 78 | 68.6 | 67 | 57.5 | *.086* |
| WCC (x109/L) (median) | **6.1** | **3.4** | **7.5** | **5.2** | ***.0021*** |
| CRP (mg/L) (median) | 22.5 | 26.5 | 24 | 49.8 | *.25* |
| Bilirubin (mg/L) | **53.5** | **54.3** | **100** | **130** | ***2.74E-07*** |
| Clinical outcomes | | | | | |
| Incidence of hospital acquired infection | 4 | 13.3 | 146 | 19.5 | *.40* |
| Incidence of kidney dysfunction | 1 | 3.3 | 96 | 12.9 | *.34* |
| Incidence of death during admission | 2 | 6.6 | 61 | 8.2 | .77 |
| 28-day mortality | 4 | 13.3 | 111 | 14.9 | *.82* |
| 90-day mortality | 6 | 20 | 179 | 24.0 | *.62* |
| 180-day mortality | 10 | 33.3 | 241 | 32.36 | *.90* |

**Supplementary Table 4.** Baseline characteristics and clinical outcomes of all patients taking long term antibiotic prophylaxis at discharge. *P*<0.05 in **bold**.

|  | Prophylaxis at discharge | %/SD | Non-prophylaxis  alive at discharge | %/SD | *P value* |
| --- | --- | --- | --- | --- | --- |
| Number | 63 | 100 % | 651 | 100% |  |
| Mean age (yrs) | 54.1 | 9.8 | 53.4 | 10.6 | *.61* |
| Male | 52 | 82.5 % | 454 | 69.7% | ***.037*** |
| Suspected Variceal Bleed | 11 | 17.5% | 94 | 14.4% | *.52* |
| Ascites | 49 | 77.8% | 419 | 64.4% | ***.032*** |
| Hepatic Encephalopathy | 13 | 20.6% | 115 | 17.7% | *.56* |
| Diagnosis of infection at randomization | 28 | 44.4% | 155 | 23.8% | ***.00034*** |
| Use of antibiotics | 40 | 63.5% | 310 | 47.6% | ***.016*** |
| MELD Score | 17.9 | 6.4 | 19.4 | 6.1 | *.45* |
| Serum Albumin (g/L) | 24 | 4.2 | 24 | 3.6 | *.41* |
| Creatinine (mmol/L) | 74 | 85.8 | 66 | 49.7 | *.074* |
| WCC (x109/L) (median) | 6.8 | 5.1 | 7.4 | 4.8 | *.99* |
| CRP (mg/L) (median) | 25 | 32.9 | 23 | 47.4 | *.80* |
| Bilirubin (mg/L) | 71 | 135.7 | 98.5 | 123.9 | *.30* |
| Clinical outcomes during trial | | | | | |
| Incidence of hospital acquired infection | 13 | 20.6% | 111 | 17.1% | .47 |
| Incidence of kidney dysfunction | 7 | 11.1% | 64 | 9.8% | .76 |
| Mortality during follow-up | | | | | |
| 28-day mortality | 3 | 4.8% | 49 | 7.5% | *.42* |
| 90-day mortality | 7 | 11.1% | 115 | 17.7% | *.19* |
| 180-day mortality | 14 | 22.22% | 174 | 26.7% | *.44* |

**Supplementary Table 5.** Baseline characteristics and clinical outcomes in patients alive at discharge that were prescribed new long term antibiotic prophylaxis or not at hospital discharge. *P*<0.05 in **bold**.

|  | Prophylaxis at Discharge | %/SD | Non-prophylaxis | %/SD | *P value* |
| --- | --- | --- | --- | --- | --- |
| Number | 35 | 100 | 679 | 100 |  |
| Mean age (yrs) | 52.0 | 8.4 | 53.5 | 10.6 | *.31* |
| Male | 29 | 82.9 | 477 | 70.3 | *.11* |
| Suspected Variceal Bleed | 5 | 14.3 | 100 | 14.7 | *.94* |
| Ascites | 27 | 77.1 | 441 | 64.9 | *.14* |
| Hepatic Encephalopathy | 6 | 17.1 | 122 | 18 | *.90* |
| Diagnosis of infection at randomization | 24 | 68.6 | 159 | 23.4 | ***2.40E-09*** |
| Use of antibiotics | 27 | 77.1 | 333 | 49 | ***.0019*** |
| MELD Score | 19.4 | 7.2 | 19.1 | 6.08 | *.90* |
| Serum Albumin (g/L) | 24 | 4.8 | 24 | 3.6 | *.81* |
| Creatinine (mmol/L) | 70 | 98.9 | 66 | 50.8 | *.38* |
| WCC (x109/L) (median) | 9 | 5.9 | 7.3 | 4.8 | *.097* |
| CRP (mg/L) (median) | 28 | 37.5 | 23 | 46.7 | *.29* |
| Bilirubin (mg/L) | 105 | 168.2 | 96 | 122.4 | *.52* |
| Mortality during follow-up | | | | | |
| 28-day mortality | 1 | 2.9 | 51 | 7.5 | *.30* |
| 90-day mortality | 3 | 8.6 | 119 | 17.5 | *.17* |
| 180-day mortality | 6 | 17.1 | 182 | 26.8 | *.21* |

**Supplementary Figure 1.** Flowchart depicting subgrouping of the ATTIRE cohort. (Note 3 patients excluded due to lack of data).


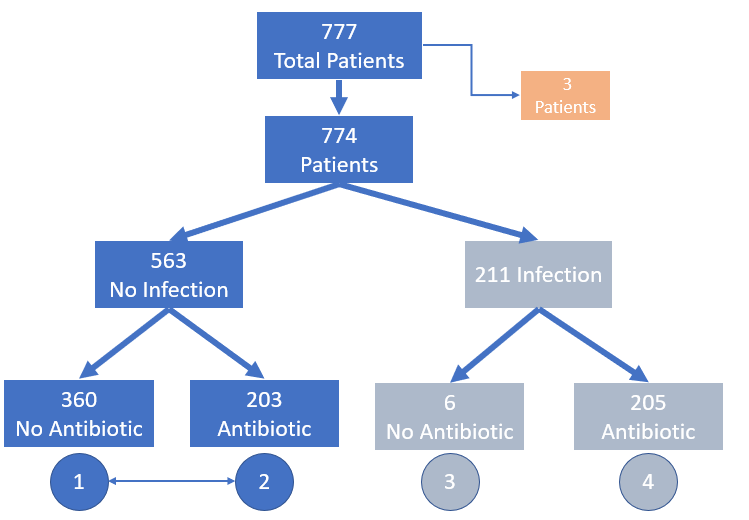


**Supplementary Figure 2.** Kaplan-Meir time to event survival analyses in matched patients taking Rifaximin or not at discharge from hospital.


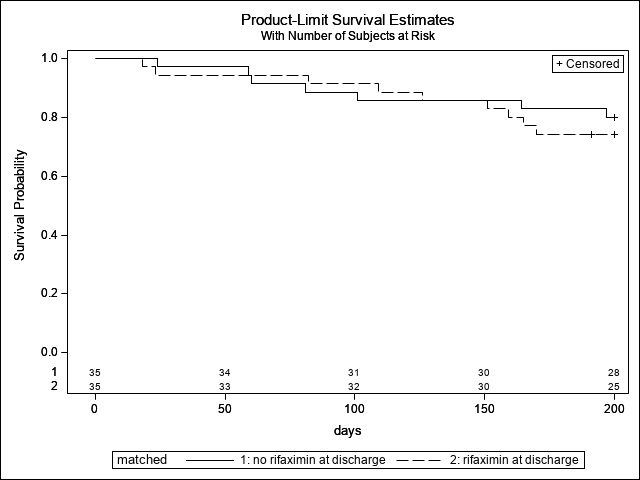

Supplement: Supplementary file 1 [file acg-118-105-s001.docx]
